# Defining conceptual artefacts to manage and design simplicities in complex adaptive systems

**DOI:** 10.1016/j.heliyon.2024.e41033

**Published:** 2024-12-09

**Authors:** Andrea Falegnami, Andrea Tomassi, Chiara Gunella, Stefano Amalfitano, Giuseppe Corbelli, Karolina Armonaite, Claudio Fornaro, Luigi Giorgi, Alessandro Pollini, Alessandro Caforio, Elpidio Romano

**Affiliations:** a International Telematic University UniNettuno, Rome, Italy; b Università Autonoma de Barcelona, Spain; c National Resarch Council - Water Research Institute (CNR-IRSA), Rome, Italy; d NBFC, National Biodiversity Future Center, Palermo, Italy; e Luiss University, Rome, Italy

**Keywords:** DSR, Grounded theory, CAS, Simplexity, Socio-technical systems

## Abstract

The concept of Simplexity has emerged several times in many discourses within different scientific domains: it somehow refers to the intertwined nature of Simplicity and Complexity. To the eye of the scientist beholder, any of these contributions renders different facets. None of those is negligible nor seems to be superior. Starting from this consideration, this paper uses a recent Design Science Research based method to develop a conceptual artefact allowing the entire realm of Complexity Sciences to unravel the Complexity of Simplexity. By moving through an in-depth systematic literature review, this research mined the nuances of Simplexity's meaning from several different research domains throughout seventy years, and then distilled those nuances. The resulting conceptual artefacts are possibly useful any time there is the need for dealing with Complexity – from the design of cyber-socio-technical systems to the analysis of protein networks – paving the way for a shared language for Psychologists, Neuro-linguists, Designers, Resilience Engineers, Biologists, and other Complexity Scientists.

## 1 Introduction

Within the intricate tapestry of scientific inquiry, the exploration of complex systems and their dynamic nature has become a focal point of paramount significance. The study of Complexity has played a central role in redefining and shaping various scientific disciplines, providing a new way of understanding and analyzing natural, social, and technological systems, emphasizing aspects such as interconnectedness, self-organization, feedback loops, emergence, and nonlinear dynamics [[1], [2], [3], [4]]. For example, in Biology, the perspective of Complexity has led to a deeper understanding of ecosystems, community assembly, gene regulation networks, and neural circuits [[5], [6], [7]]. Complexity theory has challenged the traditional view of Physics, which has traditionally been based on linear and deterministic models. A greater consideration of novel class of phenomena such as bifurcations, strange attractors associated with nonlinear and chaotic dynamics (e.g., Climate, turbulence, and swarm behavior) begot the rhizome [8] of Complexity Physics, in which complex networks, percolation and fractals find an effective application in scale-invariant and self-organizing phenomena [9,10]. In History, the assimilation of complexity theories has not only facilitated the retrospective analysis of the historians, but also enabled the formulation of projections concerning the trajectories of contemporary and future societies. Marx and Engels theorized the proletarian revolution and its seizure of power, premising this on the assertion that the progression of human history has invariably been punctuated by conflicts between social classes [11]. During the 50's of the XX century, Toynbee identified and classified 21 forms of civilization, of which four endure, all distinguished by inherent recurrent patterns [12]. Subsequently, Samuel Huntington partitioned the global landscape into discrete civilizations, formulating the paradigm of the clash of civilizations, and foreseeing that conflicts would not arise among nation-states but rather among discrete cultural entities (or civilizations) [13].

For the purposes of the present research, at its core, such exploration is weaving together Cybernetics, the study of communication and control, and its implications for Natural Sciences (e.g., Biology, Earth Science, Physics) [14,15], Cognitive Studies (e.g., Linguistics, Neuroscience, Psychology) [[16], [17], [18]], and Sciences of the Artificial (e.g., Architecture, Design, Engineering, Human Factors and Ergonomics, Medicine, Resilience Engineering) [[19], [20], [21], [22], [23], [24]]. Intriguingly, the theme of resilience takes a center stage by echoing the pioneering Holling's definition, up to the more recent by Hollnagel, which defines resilience as the ability of a system to adjust its functioning prior to, during, or following events (changes, disturbances, and opportunities), and thereby sustain required operations under both expected and unexpected conditions [25,26]. This definition delves into the systems' inherent ability to not only withstand perturbations but also to reconfigure its own functional structure and morphology. In aligning with the interdisciplinary theme of this research, ISO 9241–11:1998 enriches our understanding of Human System Integration by emphasizing the usability aspects of ergonomic requirements in various environments, which resonates with the adaptive and responsive characteristics of systems discussed in the context of Cybernetics and resilience [27].

The attentive reader will have already noticed how much it resonates with the concept of complex adaptive systems (CASs), which indeed function adapting to ever-changing environments [28]. In this advancement towards a different understanding, Complexity Theory has shown that many situations cannot be explained in terms of simple and deterministic laws. Instead, they require a holistic and multidisciplinary approach. For instance, the Theory of Networks originally emerged in Sociology, manifested as the Theory of Social Networks [[29], [30], [31]]. The study of social influence has revitalized epidemiological studies in Proteomics and, more generally, the understanding of the mechanisms underlying Information diffusion [[32], [33], [34]]. Psychology, Neurology, Linguistics, have all benefited from similar phenomena of cross-pollination (e.g., Cybernetics, Artificial Intelligence, System Dynamics) [35,36], which collectively constitute a true paradigm shift in a Kuhnian sense [37]. Complexity has challenged the traditional epistemological frameworks that considered knowledge as cumulative and static. The recognition of complex systems and the limits of reductionism have led to a greater appreciation of the nonlinear growth of knowledge, leading to the recognition of multiple truths and the importance of interdisciplinary collaboration in solving complex problems [38,39]. Constructivist approaches like Grounded Theory and Actor-Network Theory have been strongly influenced by themes of nonlinearity, multiple relationships, emergence, and have in turn nurtured all these different disciplines [[40], [41], [42], [43]]. Think of Engineering in which Complex Adaptive Systems are inspiring the design of socio-technical systems. Concepts such as Agency, the emergence of collective properties (even psychological ones like organizational cognitive dissonance), resilience, and scalability are now central in engineering infrastructure networks, artificial intelligence, and smart cities [[44], [45], [46], [47], [48], [49]].

**Fig. 1:**
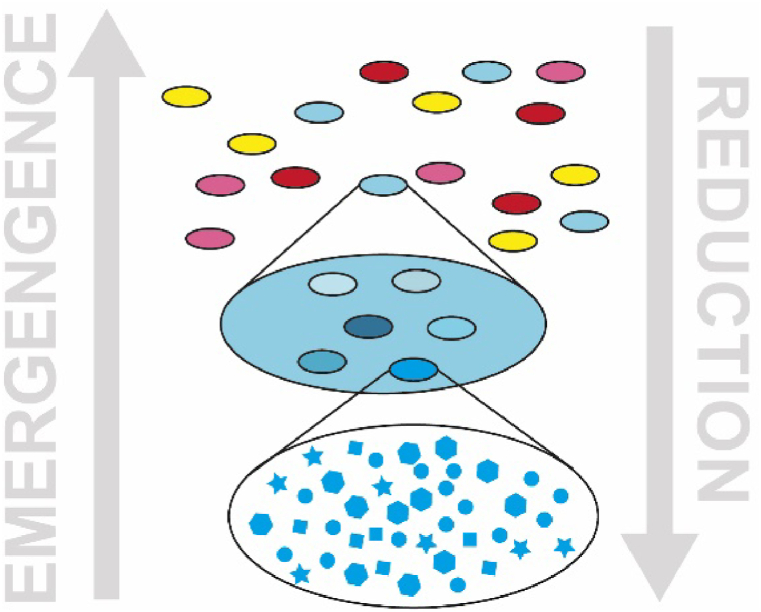
*Sketch illustrating a simplified model of reality: any depicted cone is an aggregation of unities which can be, in turn, aggregated in other unities; the grey arrows represent the opposite directions undergone by Reductionism (arrow pointing downward on the right) and Emergentism (arrow pointing upward on the left); According to Reductionism, reality can be discovered dismounting items in simpler pieces, and then studying those pieces' behavior; the opposite holistic approaches reveal that Reductionism misses some important phenomena called* emergent.

It is difficult to account for all the fields that have adopted such perspective. The realm of Complexity sciences is continually evolving. Moreover, interdisciplinarity often leads to the emergence of new areas of study. From this straightforward observation, as well as the findings presented above on the constructivist perspectives envisioned by contemporary Science, arises the need for building a lexicon that corresponds to the semantics we perceive in our research paths. The traditional physical-mathematical approach is fundamentally reductionist (see Fig. 1), therefore its lexicon cannot fulfil our need, but our discourse can embrace it.

Reductionism identifies a simple idea (the so-called *natural law*) as the underlying mechanism capable of explaining many phenomena. However, these simple objects are an abstraction of too many aspects of reality. In fact, it is not a coincidence that reductionist theories focus on aspects and systems that are at very distant scale of the space-time continuum (e.g., Quantum Physics and Einstein's Relativity) or only on some extremely simplified aspects at our dimensional scale (e.g., material points, rigid bodies, elastic systems) [50,51].

Albeit successful in the past, this approach is outright unsuitable for those systems inhabited by human activities [52]. Socio-technical systems are a notable example which require a holistic approach to account for so-called emergent phenomena [53,54]. The contemporary approach rejects the idea that reductionism can offer a comprehensive explanation of reality on its own, and requires the opposite approach, i.e., the emergentist one, to be integrated. However, this requires the integration of associated semantics as well. Hence the need for an appropriate lexicon.

Reality can be semantically arranged on a Simple-Complex axis: there are *simple* natural laws that give rise to complex phenomena, just as from complex systems (e.g., turbulence, fractal structures), broadly predictable behaviors can be discerned due to their simple regularities. As authors who mainly focus on socio-technical systems, we feel that Emergentism has not contributed to the discourse of Simplicity and Complexity with the same depth thus far. And, from a flipped perspective, holistic approaches have encountered similar patterns of Simplicity and Complexity that need to be arranged, if not in the same axiological dimension of Simple-Complex, then within an appropriate structure of meaning. Various authors from distinct disciplinary areas have independently explored the same Simple-Complex axis, questioning a concept capable of integrating this dichotomy that they named *Simplexity* [[55], [56], [57], [58], [59], [60], [61], [62], [63], [64], [65], [66], [67], [68]]*.* However, these scholars highlighted different aspects.

A unified definition of Simplexity must be firmly provided, as a starting point for an articulated theory of Complexity and Simplicity capable of representing phenomena in a way that is no longer just intuitive when dealing with socio-technical systems, removing their coat of vagueness. Other Complexity scientists experience the same need, but for us engineers of socio-technical systems, the purpose is not purely speculative; we need it as a design tool. For example, as we explore the integration of human-centered design into complex systems, it's essential to consider how such approaches have been shown to enhance both system functionality and user engagement across various fields, including technology and education [[69], [70], [71]]. In this context, we refer to constructs as particular cognitive artefacts in the sense of Herbert Simon and as understood in Design Science Research (DSR): objects resulting from human cognition from which other symbols, algorithms, models, principles, and tools can be derived [24,72,73].

This research aims to design such cognitive artefacts in the sense of DSR, precisely to provide studies of socio-technical systems with the power of a specialized language that redefine the duality of Complexity/Simplicity. Through a constructivist approach, a Simplexity-based set of constructs is proposed in this article following a recent DSR method [74]. Using a literature analysis based on the P.R.I.S.M.A. methodology, 86 articles and 3 books related to the concept of Simplexity were examined.

The remainder of the article continues as follows: §2 Refers to the methodological framework adopted in the methods described in §3; §4 and §5 illustrate the results and discuss them, respectively. Finally, Section §6 draws some conclusions.

## 2 Methodological framework

The method proposed by Akoka et al., in 2023 is well suited for the purpose of this research, since it provides structured directions about how a design science project should be conducted, following suggested research strategies in order to initiate, develop, and articulate a novel knowledge contribution [74]. Those authors proposed a particular construct (i.e., conceptual artefact) called *path of knowledge* allowing for capturing the dynamic nature of knowledge contributions. The following paragraphs rely deeply on concepts of this method. Please refer to the original article for more details.

### 2.1 Preliminary concepts

#### 2.1.1 Study goal and scope

According to Akoka et al., a generic DSR study can be defined by its goal and scope [74].

The knowledge goals can be.

1. *Definitional*: to collect concepts, terms, and classifications providing the basis for expressing other knowledge without making claims about reality.
2. *Descriptive*: to achieve knowledge that describes and analyzes existing or past reality, stating true claims without explanations or predictions.
3. *Explanatory*: to answer “how” and “why” questions, offering understanding by explaining causal relationships and underlying mechanisms.
4. *Predictive*: to predict outcomes based on underlying factors without explaining causal relationships, focusing on accurate predictions.
5. *Explanatory* and *Predictive*: to provide both explanations and predictions, describing causal relationships and predicting outcomes.
6. *Prescriptive*: to provide models and methods that guide problem-solving, offering guidelines for practical application.

Differently, the knowledge scope represents the extent of the study, and it might assume two values: it can be general (*nomothetic*) or specific (*idiographic*).

#### 2.1.2 Knowledge types

A knowledge type is obtained considering concurrently knowledge goals and scope. There are eleven possible different knowledge types. The interested reader can refer to original table 2 in Ref. [74].

#### 2.1.3 Paths of knowledge as digraphs

*The nodes of a path of knowledge.*

A DSR study progresses throughout several knowledge moments – knowledge type contributions occurring in different steps – which together form a path of knowledge. In other terms, a path of knowledge can be seen as direct graph whose nodes are different knowledge moments which, in turn, can be one of the above mentioned eleven knowledge types [74].

*The edges of a path of knowledge.*

Even the edges of the path of knowledge digraph can be labeled, not only the nodes. This makes explicit the semantics behind the relationship between two consecutive knowledge moments (Please refer to table 4 of [74]).

**Fig. 2:**
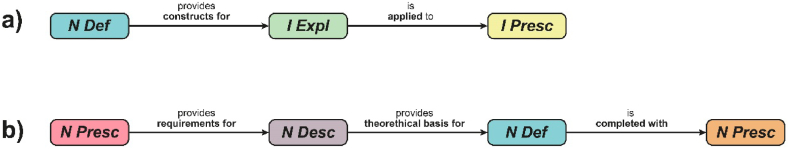
Two exemplary paths of knowledge. a) passes through three knowledge moments, while b) through four. The labels (i.e., the knowledge types) chosen for the knowledge moments are N Def, I Expl, I Presc for a) and N Presc, N Desc, N Def, N Presc for b). Each edge is labeled, making explicit the semantics among the different knowledge types.

For Example, Fig. 2 shows two exemplary hypothetical paths of knowledge: the path in Fig. 2-a passes through three knowledge moments, while the path in Fig. 2-b passes through four. Both knowledge moments and edges are labeled accordingly (see tables 2 and 4 of [74]).

#### 2.1.4 Paths of knowledge types and DSR-research strategies

##### 2.1.4.1 Research strategies dimensions

Starting from the work of Iivari [75] who listed the different dimensions in which the DSR knowledge production strategies are articulated (i.e., kind of knowledge production; Research and evaluation methods; resources needed), Akoka et al. drew up 32 possible different types of paths of knowledge which were further classified into seven research strategies which are synthesized in Akoka (see table 8, and table 9 of [74]) as well as in Table 1.

**Table 1:** DSR strategies resources dimension classification.

#	Strategy Name	Aim	Expertise needed	Involvement of a client	Time
**1**	Strategy 1a	Build and instantiate artefact	Disciplinary	Optional	*Quantum sufficit*
**2**	Strategy 1b	Build and instantiate artefact, adding to definitional or descriptive knowledge	Disciplinary.	Optional | Participation of practitioners in evaluation	*Quantum sufficit*
**3**	Strategy 2a	Build and generalize from instantiations	Multi-disciplinary	Required	Projects occur over an extended period of time
**4**	Strategy 2b	Build and generalize from theory-grounded instantiations	Multi-disciplinary	Required	Projects occur over an extended period of time
**5**	Strategy 3	Hypothesize and test propositions	Disciplinary	Not required	*Quantum sufficit*
**6**	Strategy 4a	Build design theory	Disciplinary | Multi-disciplinary	Optional	Additional timeframe for building and evaluating artefacts
**7**	Strategy 4b	Build design theory, adding to design-relevant explanatory/predictive theory	Disciplinary | Multi-disciplinary	Optional	Additional timeframe for building and evaluating artefacts

##### 2.1.4.2 Guidelines based on paths of knowledge types and strategies

Akoka et al. finally proposed guidelines to be followed in order to conduct a DSR project and adding a knowledge contribution [74]. G1: the focus is on initiating a DSR project by thoroughly examining the context and selecting an appropriate strategy aligned with the characteristics of the project. This foundational step ensures that the research is rooted in a relevant approach from the outset. G2 emphasizes the importance of building initial knowledge. At this stage, starting from the chosen strategy, the researcher should analyze the prototype and relevant exemplars to uncover potential paths that lead to various types of knowledge. This examination of existing examples within the context of the chosen strategy enables a structured yet exploratory approach to identify new knowledge pathways. As research progresses, G3 provides guidance on advancing ongoing research by matching paths and exemplars. Here, the researcher should reassess the chosen strategy in light of the research's progression and attempt to align the path traveled with the exemplars related to this strategy, thereby revealing the next possible steps. This matching process is essential for identifying logical continuations within the current strategy framework. The guideline G4 addresses situations where ongoing research requires a shift. When matching the current path with the prototypes and exemplars of the chosen strategy no longer yields further alternatives, it may be necessary to adopt a new strategy. In cases where a more disruptive approach is warranted, G4 advises researchers to explore the possibility of conducting research that bridges strategies, effectively drawing connections or edges between them to uncover novel insights. This guideline underscores the importance of flexibility and adaptability in navigating complex research trajectories.

By choosing a strategy the research is steered toward a certain path: «Each strategy produces diverse knowledge types, requires different resources, and resorts to different methods (the three groups of dimensions). Evaluation and publication standards differ» [74].

Therefore, once the research question and the available knowledge exemplars have been identified, it is a matter of selecting the most appropriate guideline. The related strategy will define the various knowledge moments and, thus, the path of knowledge to be followed.

### 2.2 Simplexity's path of knowledge

The research group followed the guideline G1 (Initiate a DSR project) and, considering RQ1 (i.e., How can we represent the contributions of design science research, recognizing its dynamic, pluralistic, and contextual nature?) and RQ2 (i.e., How can we characterize, and learn from, representative knowledge contributions in prior research and classify them into useful strategies for future projects?), then chose the Strategy 1b (i.e., Build and instantiate artefact, adding to definitional or descriptive knowledge), whose application to our research project will be thoroughly explained in the next section.

## 3 Adopted methodology steps

The research group adopted the DSR standing point and chose to structure its research by following the theoretical framework described in section 2. The overarching adopted methodology is summarized in the following steps (Table 2):

**Table 2:** The overarching adopted methodology.

Step	Description
*4.1 Applying the DSR theoretical framework*	The adopted methodology starts by applying the theoretical framework introduced in section 2. Guideline G1 was followed and then Strategy 1b was chosen.
*4.2 Applying a P.R.I.S.M.A.-based protocol for systematic reviews*	Major Knowledge contributions returned by applying Strategy 1b are methods and constructs. P.R.I.S.M.A. was identified as an ideal methodology to follow for collecting required initial data.
*4.3 Reading and categorizing documents*	The documental sources were read and categorized through several dimensions for identifying relevant preexisting constructs and theoretical bases for the subsequent steps.
*4.4 Literature Matrix construction*	The dimensions defined in step 4.3 are used to build a literature matrix (i.e., a matrix in which each row is a document from the P.R.I.S.M.A. literature review, and the columns represent the document's features).
*4.5 Implementing Grounded Theory Methodology*	Some researchers implement Grounded Theory to saturate semantics and integrate concepts.
*4.6 Focus Group*	The whole research team discusses the different elicited nuances of meaning and, after reaching unanimity, proposes novel integrated constructs pertaining Simplexity-Complexity axiology.
*4.7 Validation of the obtained constructs*	The new constructs must be validated by experts and professionals.

The steps constituting the methodology are distinguished only in theoretical terms; in fact, the research process is never linear: these steps, here presented as consecutive, were partially overlapped both in time and in their conceptual development. However, for the sake of clarity, each step is detailed separately below.

### 3.1 Applying the DSR theoretical framework

The research project started by assuming the theoretical framework introduced in section 2. Guideline G1 was followed and then Strategy 1b was chosen since this latter expects definitional knowledge as a major contribution. Since its declared starting point is knowledge from natural and social science, an extensive literature review was identified as a primary moment of knowledge. P.R.I.S.M.A., a systematic literature review methodology designed for evidence-based medicine scientific projects [76], is assumed to be one of the most complete.

Even though the resources dimension for strategy 1b prescribes a disciplinary research team, the high heterogeneity of the scientific domains addressed by the documents attained (see Table 1), the research project was deemed to require multidisciplinary competences. Thus, the team included eleven researchers with mixed background and expertise: two in Industrial Engineering and Operations Management: one is an expert of Socio-Technical Systems and Resilience Engineering, the other one is an expert in Optimization and Simulation; two are Psychologists: one expert in Cognitive Processes and Technologies and the other in Educational Psychology and Learning Theory; one is a Geoscientist; one is an expert in Brain Sciences and Computational Neuro-science; one is an Information Technology scientist expert in Computational Linguistics; the remaining ones are an Architect specialized in Human-technology Interfaces and Ergonomics, an Anthropologist, and an Historian. A Biologist was later deemed necessary to be added to the team because of the nature of the covered topics. Beyond this formal categorization, many of the research team share common interests in DSR and Complexity Sciences.

**Fig. 3:**
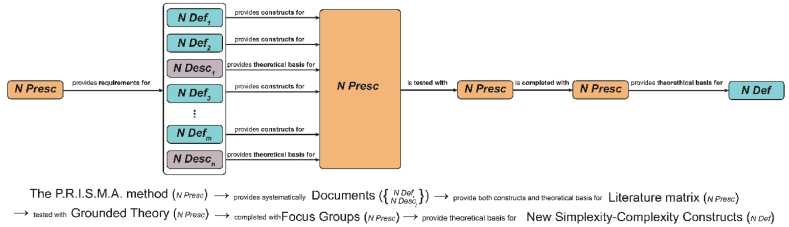
The Simplexity Concept path of knowledge. An initial nomothetic prescriptive knowledge moment (the P.R.I.S.M.A. method) provided requirements for systematically retrieving several nomothetic definitions and description of Simplexity (witnessed by the documents); these latter provided preexisting constructs and theoretical basis to be included in a nomothetical prescriptive tool – the Literature Matrix. Such artefact was tested with Grounded Theory method and then completed with a focus group meeting (both N Presc). The next knowledge moment is represented by the final sets of constructs which, as required, are nomothetical definitions.

Fig. 3 depicts the path of knowledge followed in this DSR project to produce novel knowledge artefacts contributing to the study of complex adaptive systems such as socio-technical ones. The knowledge path starts from a nomothetic prescriptive knowledge moment (e.g., the application of the Preferred Reporting Items for Systematic Reviews and Meta-Analyses, P.R.I.S.M.A.) which provides each document belonging to the corpus. Even though it has been represented as a monolithic object (they happen in the same knowledge moment), it is composed of two kinds of knowledge types. Some articles describe natural phenomena in which some Simplexity phenomenon occur, which is typical of nomothetic descriptive science (*N Desc*). The others directly provide a definition of Simplexity (*N Def*). Indeed, there are some articles belonging to the corpus identified with P.R.I.S.M.A that might be at the same time *N Def* and *N Desc*, but this would not change the nature of the path. The only difference stays on the edges: while the *N Def* knowledge type provides constructs, the *N Desc* provides theoretical basis for the subsequent knowledge moment, which is the built literature matrix (see supplementary materials).

The literature matrix is a general conceptual artefact (*N Presc*) which prescribes the data collection and steers the Grounded Theory stage (*N Presc*) (see § 3.5). The information elicited during this knowledge moment has been completed in the subsequent focus group (*N Presc*), which in turn provides theoretical basis for the novel definition of the Simplexity-Complixity (§ 5.4) related conceptual artefacts (*N Def*).

### 3.2 Applying a P.R.I.S.M.A.-based protocol for systematic reviews

The purpose of this phase was the identification of relevant sources to represent already available knowledge on Simplexity. The present systematic review adhered to the guidelines and principles set forth in the 2020 Preferred Reporting Items for Systematic Reviews and Meta-Analyses (P.R.I.S.M.A.) statement [76,77]. A comprehensive search was conducted within the Scopus database to identify relevant studies ever published, i.e., by searching from the oldest occurrence of the concept addressed in literature. Furthermore, any additional studies not initially identified by Scopus but aligned with the objectives of this systematic review were added and to the reference list. Scopus scientific database was deemed as a trustworthy and adequate source of documents. Some estimates suggest that Scopus coverage is over 86 % [[78], [79], [80]]. The search query submitted on June 23, 2023 was: TITLE-ABS-KEY (Simplexity). In our literature review, we deliberately concentrated on articles explicitly referencing the term “Simplexity”. It is imperative to acknowledge that, when examined within the framework of various cultural contexts, the concept of Simplexity can be significantly influenced by linguistic and societal factors. Within specific societal settings, individuals may readily embrace the idea of “Simplexity” without explicitly categorizing it as such. This may also be reflected in the literature and consequently some authors may have dealt with the topic of Simplexity without explicitly classifying it with this term.

The search returned 86 documents. Two of these were not found. After performing the subsequent stages of the review process, five more documents were collected (two articles and three books) for a total of 89 documents (55 articles, 6 reviews, 6 editorials, 8 conference papers, 1 online document, 8 book chapters, and 5 books). It must be noticed that three different retrieved occurrences referred to different language versions of the same book [56,81,82], therefore the above document count already considers them to be coinciding. Our inclusion criteria were primarily focused on original peer-reviewed research articles. To ensure the highest possible conceptual robustness, we conducted a review of analysis units following the track recommended by the P.R.I.S.M.A. methodology for systematic reviews [76]. This method simplifies identification and minimizes bias in the interpretation of articles.

### 3.3 Reading and categorizing documents

During this step the contributions gathered were distributed among the researchers according to their field of expertise and personal inclinations. Some documents were read and analyzed by at least two team members (cfr. § 3.5). The team members were requested to categorize the documental corpus by the application domain, to report the definition of Simplexity (if present) given, as well as to provide a personal interpretation of the text. In addition, the researchers had to refer, for any documents analyzed, if there were references concerning Simplexity. This stage was important to elicit the cultural background in which the concept of Simplexity developed and evolved, as well as to identify the eminent contributors to its varieties of meaning.

### 3.4 Literature matrix construction

The data collected in the previous stage, combined with bibliographic one, constitute the material used in constructing the literature matrix – a prescriptive research artefact – typically in use during analysis of a documental corpus [83]. The literature review (provided in the supplementary materials) presents the attained documents in the rows, while the reported columns are Doc_ID; Authors; Title; Year; Abstract; Keywords; Document Type; Simplexity References; Attained; Summary; Domain; Definition; Interpretation.

### 3.5 Implementing Grounded Theory methodology

In this step, the research team followed Grounded Theory prescriptions [41]. The literature matrix documents were subjected to open coding.

From initial document-level coding, each researcher moved to word-by-word coding with the aim of fragmenting the corpus toward a single meaning unit level. At this point the researchers who read the same documents compared their interpretations – surely different due to the unique scientific background and epistemic perspective of the single researcher; this process allows for further emergence of suggestions to be reprised in the following coding phases. It was necessary to juxtapose the ideas elicited with the conceptual background that produced those thoughts. Frequently, researchers encountered some unprecedentedly known concepts, mainly in Biology – e.g., *Umwelt* disclosed by von Uexküll, [84,85]. To categorize those concepts, the initial ten persons research team expanded to include a Biologist.

The focused coding stage started with the rough conceptual categories labeled in the previous coding stage but not yet saturated. It was possible to gather many concepts in some clusters during this stage. Few of them were turned into higher-level concepts. In addition, the synthesis process, some proto-categories, capable of addressing as many literature items discovered as possible, were identified by likeliness or by semantic importance; concurrently, these proto-categories were mutually linked and connected to the sub-categories identified earlier. This process is known as axial coding. Once the semantic units were consolidated, the Grounded Theory researcher should assign a meaningful name to them.

Subsequently Theoretical Coding started: the core category (i.e., Complex Simplicities), the concept that unifies the categories and justifies the subtle differences within each paper, emerged. Such concept is an analytically powerful core category, capable of integrating all the different perspectives, ramified, complete and with great explanatory power, as prescribed by Grounded Theory. Complex Simplicities is therefore the conceptual artefact the research project was looking for. It had only to be elicited from the ideas contained in the documentary corpus. The Complex Simplicities core category brings together other constructs (e.g., Simplicity; Complexity; Complexity Simplicities: Simplexity, Complicity/Complixity; Compression of Complexity; see §5).

### 3.6 Focus group

A final focus group meeting was held to summarize the previous steps’ results. Throughout the discourse, the participants brought many different perspectives to the table. The novel conceptual artefacts were considered ready only when the researchers totally agreed on their definition.

### 3.7 Validation of the obtained constructs

The concepts were initially validated among researchers and then discussed with other academic colleagues informally. To make the work more reliable, we took this last step to challenge peer criticism. The research team expects to be able to apply the concepts obtained in upcoming knowledge contributions and other types of artefacts as soon as possible. However, a more extensive validation can only be achieved with the contribution of those working in DSR, Resilience Engineering and other disciplines potentially affected by the Simplexity concept. Parallel and independent research has produced an article of profound relevance. This article (see §5.5) was published in the same month as the first submission of our own work (October 2023) as a partial validation of the present work [86].

## 4 Results

The documentary corpus appears to be mainly composed of articles and books (see section 3.3) and is rather fragmented across different sources. Full results of the Literature Matrix are provided in the supplementary materials.

Focusing only on journal publications, *Language Sciences* gathers the highest number of articles (6) – all of them appearing simultaneously due to a special issue titled “Simplexity, agency, and language”. Then *Chinese Semiotic Studies* (5). Following that there are: *Discrete Mathematics*, *Geographical Analysis*, *Kybernetes,* and *Management Learning* with 3 articles each. *Biosemiotics* has 2 articles. Finally, there is one article each for *Actualite Chimique*, *Angewandte Chemie*, *British Journal Of Management*, *Computational Geometry Theory And Applications*, *Computers And Education Artificial Intelligence*, *Computers And Mathematics With Applications*, *Computers In Industry*, *Corrosion*, *Disegno*, *Doklady Earth Sciences*, *Electronic Notes in Discrete Mathematics*, *Engineering Management In Production And Services*, *Entropy*, *European Journal Of Combinatorics*, *European Journal Of Mineralogy*, *European Management Journal*, *Evolution Psychiatrique*, *Frontiers In Artificial Intelligence*, *Human Relations*, *International Journal Of Inclusive Education*, *Journal Of E Learning And Knowledge Society*, *Journal Of Functional Analysis*, *Journal of Geology*, *Journal Of Geosciences Czech Republic*, *Journal Of Neuroscience Methods*, *Knowledge And Process Management*, *Mineralogical Magazine*, *Minerals*, *Philosophies*, *Revue De Synthese*, *Structural Chemistry*, *Studia Romanica Posnaniensia*, *Synergies Europe*, and *Synthese*. Just from this list of journals it can be inferred that the concept of Simplexity, although not yet standardized, is shared by many scientific disciplines, seemingly distant in their interests. In fact, we are facing a dual mechanism of semantical evolution: on one hand, we observe a phenomenon of *convergent evolution* (i.e., the emergence of a similar concept in different cultural niches due to similar conditions of opportunity); on the other hand, we witness a process of transmission from one domain to another, almost a *memetic contagion* [87,88], which, through repeated tiny variations and/or shifts in meaning, transform (and consolidate) certain aspects of the concept over time. The first mechanism has created over time three independent meanings, while the second has favored one, which is deeper and more adaptable, hence it has ended up colonizing different domains. The result of this evolutionary process is five aspects of Simplexity, which are subsequently referred to as *nuances of meaning* (Table 3).

**Table 3:** The resulting meanings of Simplexity core concept as results of convergent semantical evolution through the ages of literature are presented as columns. Possibly a meaning can convey several nuances, which are in the rows. For meaning II and III there is only a nuance each. Meaning III is starred since it shall be disregarded.

Simplexity Concept		Meanings as results of convergent semantical evolution		
		I	II	III ∗
**Nuances of meaning**	1	*Mineral*		
	2		*Geometric*	
	3	*Organizational*		
	4	*Epistemic*		
	5	*Biological*		
	6			*Board Game*

**Fig. 4:**
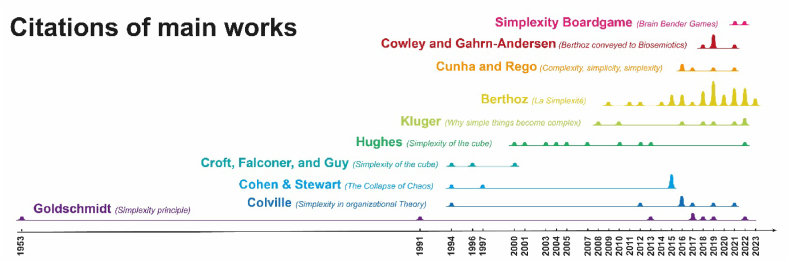
Evolution in time of the citations limitedly to the documentary corpus; the different colored lines account for the Authorities (i.e., authors that introduced innovation in Simplexity conceptualization or brand-new application in a different domain). The visual accounts for the entire lifespan for each authority in terms of citations; the hills are proportional to the citation hits for that year. Goldschmidt (purple) is a long-lasting authority having rare low hits; Berthoz (yellow) authority exhibits a shorter and more recent lifespan frequently cited by other authors also coming from different domains (cfr. Fig. 5). The colors used in Fig. 5, Fig. 6 are for the most the same, keeping partially track of the elicitation process followed by the research team.

**Fig. 5:**
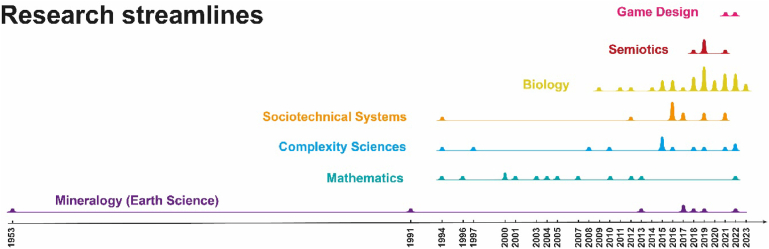
Evolution in time of the research streamlines expressed by the documentary corpus. The guiding principle for attributing the streamline is the belonging domain regardless of the citation. For example, Berthoz (Biology, yellow) in his book cited Goldschmidt (Mineralogy, purple), but since it completely renovated it, it was not accounted for. However, many documents can be traced back to the same original authority, e.g., all the documents referring to Goldschmidt concern Earth Science related contributions (purple). The colors mostly follow the same scheme of Fig. 4.

**Fig. 6:**
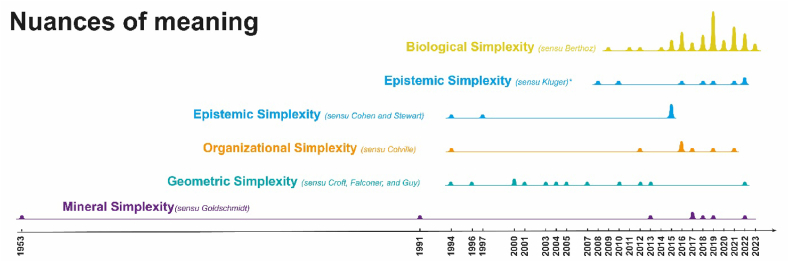
Evolution in time of the collected Simplexity concept nuances of meaning (see Table 3). As before, colors follow the research process (see Fig. 4, Fig. 5). Whenever a nuance has deemed irrelevant was disregarded (cfr. Game design in Fig. 5). The corresponding original Authority's contribution to the nuance is reported in brackets). For example, the Simplexity Concept's 4th nuance of meaning I (cyan) is split according to the two major authorities.

Due to the adoption of this perspective, it doesn't make much sense to analyze the corpus from the point of view of the involved nations (United States, Italy, Denmark, France, U.K., and the Russian Federation being the countries with the highest participation). Instead, it is worth reconstructing the stages of this memetic diffusion through a diachronic reading of the comparative analysis of the documentary corpus, using as a benchmark the article (and therefore the author) that introduced or significantly modified a particular nuance of meaning. This analysis was conducted, firstly, by reading the articles (see §3.3) and their structuring in the literature matrix (see §3.4) – a true *literary inscription* in the Latourian sense [89]. Subsequently, the concepts were refined through Grounded Theory (see §3.5 and §5). The colors in the figures (Fig. 4, Fig. 5, Fig. 6) bear witness to this refinement that occurred in the synthesis process [[89], [90], [91]].

### 4.1 Evolution of the citations

Firstly, we identified the documents in which the concept of Simplexity is proposed and when such proposals are accepted and cited (Fig. 4). For example, the very first formulation of the Simplexity dates back to 1953 by Goldsmith [60]. This very first definition persists over a long timeframe (extending up to 2022), although it is cited very rarely. The article deals with Mineralogy, a very specific and rather limited field compared to the documentary body. As often happens, however, the concept expressed by Goldschmidt and synthesized by the term Simplexity is quite evocative and appealing to other researchers from different research areas. This is the case with Berthoz himself, who, when discussing Theoretical Biology, explicitly states that he is referring to the term Simplexity used by Goldschmidt, redefining it for his own use [56]. Therefore, we observed a real straying into another discipline. On the other hand, there was, however, a radically different sense of Simplexity associated with polytopes in Mathematics, which are useful in Optimization Theory and Topology [92]. In this second case, we cannot interpret simultaneous coexistence as contamination but rather as a genuine case of polysemy.

### 4.2 Evolution of research streamlines

To highlight analogies and differences, we transitioned from a citation-based documentary analysis to an analysis based on research streams (Fig. 5). Seven main research streams emerge: the most ancient one pertains to Mineralogy and, more generally, Earth Sciences [61,61,93]. Roughly contemporaneous (within the corpus) are the streams related to Socio-Technical Systems [57,65], Complexity Epistemology [94,95], and Mathematics [96,97,97,98]. The articles in Mathematics discuss Simplexity as a generic attribute of a simplex (i.e., a polytope in n-dimensional space). This concept has its origins in the distant past, potentially predating the inception of the temporal span encompassed by the corpus. Subsequently, the Biology stream emerged first (and one might say, proliferated), followed by the Semiotics stream – a result not surprising given the various mutual contaminations between the two disciplines (e.g., Biosemiotics, Theoretical Biology) [59,62,[99], [100], [101], [102]]. The most recent stream is also the most unrelated (e.g., Game Design) [103,104]. Indeed, it is a case of homonymy; Simplexity, in this case, is actually the commercially adopted name for a board game – hence irrelevant to our purposes – although it can be considered yet another instance of convergent evolution. From a purely lexical perspective, the term “Simplex” means “folded only once”, while “Complex” means “folded multiple times”. Therefore, Simplex is an archaism for “simple” evidently convenient when one wishes to denote something that is straightforward but not simplistic.

There are documents that, despite not being reviews, simultaneously cite works belonging to different disciplines because they can still be related to analogous semantic nuances [105].

### 4.3 Evolution of nuances of meaning

The analysis proceeded to group documents by semantic proximity and then to prune irrelevant meanings (i.e., meaning III in Table 3). This resulted in 5 clusters of interest (Fig. 6). It should be noted that the Complexity Sciences stream (in cyan) is divided into two different ideas: one according to Stewart and Cohen (2000) and the other according to Kluger (2008). The latter is more of a collection of examples of phenomena that can be attributed (according to the author) to the concept of Complexity, and thus, Simplexity. It is a popular science book that has not contributed much to the discussion, cited more for completeness than for significance.

Leaving aside the other species of convergent Simplexity (e.g., digital twins and board games) that have been disregarded in subsequent analyses, among the six resulting nuances, there is no clear drift in terms of meaning. It is something that articulates – we could say – “in a complex manner” the dichotomous axis of Simple-Complex. In more detail, the more specific nuances of meaning gathered are.

• **Mineral Simplexity**: Several documents, with Alain Berthoz's work (“La Simplexité”) prominently among them, cite a document (dated 1953) that initially was not included in the documentary corpus but was added as a source later on. This document is “A 'Simplexity Principle' and Its Relation to 'ease' of Crystallization” by Julian R. Goldsmith [60]. The article represents the earliest known usage of the concept of Simplexity as a simplifying property of a complex system. Specifically, it describes how the process of crystallization can be eased (and thus simplified) by entropic disorder – a measure of structural Complexity – referred to as Simplexity. This particular nuance of meaning certainly transmitted over time within its original discipline. However, it would find more fertile ground when embraced in Biology as a similar property of living systems, where they utilize their own Complexity to simplify their interaction with the world. All the nuances of meaning I budded from this Goldsmith's article (see Table 3). Other notable examples of articles belonging to this research stream are [[106], [107], [108], [109], [110]]. Reference is made to the Literature Matrix in the supplementary materials for all other cases.
• **Geometric Simplexity**: this nuance of meaning is strictly related to Optimization Theory and Topology. As already mentioned above, here the term “Simplex” refers to a n-dimensional polytope, named in this way since it is the simplest possible polytope in that given R^n^ space. This concept belongs to the realm of Geometry, and it is antecedent to the definition given by Goldsmith in 1953. Therefore, it represents a nuance of meaning II (Table 3). Differently from meaning III (which is completely irrelevant to the Simplexity discourse), meaning II still can be understood in terms of dialogical tension between Simplexity versus Complexity [[111], [112], [113]]. Berthoz himself in a dedicated paragraph of his “La Simplexité” writes about the concept of geometrical simplex, showing off how this particular nuance of meaning resonates with the biological one.
• **Organizational Simplexity**: it is the nuance of meaning I embracing all the contributions coming from Organizational Theory and Complexity Sciences applied to the study of socio-technical systems (e.g., Resilience Engineering). Certainly, since many concepts regarding collective agency and sensemaking processes are borrowed from Psychology and Cognitive Studies, there are many semantic contaminations. Therefore, several documents might be classified as contributions to Organizational Simplexity as well as to Biological Simplexity. As an exemplar contribute can be cited “Complexity, Simplicity, Simplexity” by Miguel Pina e Cunha and Arménio Rego [114]. The authors carry on the legacy of organizational studies in Cybernetics, reframing the Ashby's law of requisite variety and the Beer's Viable System Model [[115], [116], [117]]. Beyond addressing the usual notion of Simplexity as a synthesis of Simplicity-Complexity dualism, they suggested novel insights analyzing how Complexity and Simplicity coexist and coevolve. For example, the two authors noticed how growing Complexity coevolves with increasingly sophisticated administrative/formal systems seeking to reduce uncertainty. Therefore, growing organizations become more complex and this fosters inner specialization. A paramount authority in the organizational studies is Weick which is cited over all the corpus [57,118,119]. He reasoned about the need for envisioning Complexity in thinking and sense-making. “Complicate yourself” He used to recommend to managers [114]. In this nuance of meaning is also present the concept of Complexity as a cumulative by-product of organizational changes that over time weave complications often invisibly. This is also a concept recently embodied by Resilience Engineering's Organizational Drift [120,121]. This concept is important since it underlies the recurrent idea that little simple modifications can lead to emergent complex behaviors at system's level.

Artefacts like organizational processes are usually designed to increase Simplicity, but sometimes they produce undesired Complexity but, more interestingly, there is also an unintentional Simplicity: in the long-run, success in winning organizations (we would say “fitted”) leads to simplification which, in turn, may diminish the organization's “peripheral vision” and, hence, favor exploitation over exploration [114]. This fitting pattern ultimately lessens the anticipation ability of the organization. Another crucial point raised by this nuance of meaning is the need to depart from a traditional mechanistic view associated with Newton in favor of a fractal perspective of organization [122,123]. Such a perspective may highlight the interplay between Simplicity and Complexity occurring at various levels of organization. Furthermore (and this adds meaning and value to the present research), researchers are often encouraged to explore the promising avenues of the Simplicity/Complexity interrelationship concerning certain traditional management concepts (e.g., routines, repetition, variability, consistency) through the lens of Simplexity. This is because Complexity depends not only on the object but also on the language used to describe that object. Possessing an appropriate vocabulary for Simplexity can lead to a better understanding of socio-technical systems as self-adaptive complex systems [124].

• **Epistemic Simplexity**: this cluster has proven to be one of the most interesting for the purpose of the current research. This nuance also falls under the meaning I. The fundamental contributions, we could almost say the two main theses of this nuance of meaning, have been put forth in two of the three books included in the corpus. We are talking about “The Collapse of Chaos” by Jack Cohen and Ian Stewart, and “Simplexity: Why Simple Things Become Complex (and How Complex Things may be made simple)” by Jeffrey Kluger, respectively [94,95]. The second contribution is often cited, although it never contains a definition of Simplexity, nor can its meaning be inferred from reading the text. It is a popular science book that generically describes mechanisms sometimes attributable to the actions of Chaos and at other times to the non-linearity of systems, but it lacks a clear unified vision. It has been included for completeness and due to the numerous citations, as mentioned earlier. On the contrary, the book by Cohen and Stewart gathers and synthesizes some epistemological themes of Complexity Science, albeit doing so through narrative devices – a technique often used by authors to introduce concepts that are not necessarily easy to grasp, e.g., in Ref. [125]. We found, well-articulated, some questions posed by Morin, Heisenberg, and Latour [4,126,127]. The core of the argument revolves around the axes of Simplicity/Complexity, Reductionism/Emergentism, Content/Context, Deduction/Induction, and the perspectives of Newton/Darwin. In a clever manner, the two authors propose pairs of contrasting constructs: Simplicity-Complexity and Simplexity-Complicity. It's a well-constructed articulation that deserves further development (see §5.4). The triad of articles proposed by Maurice Yolles and Gerhard Fink, titled “A General Theory of Generic Modelling and Paradigm Shifts,” can be attributed to this nuance of meaning (although equally, it could be part of articles related to Organizational Simplexity as well as Biological Simplexity) [[128], [129], [130]]. This work is conceptually unified and can be attributed to the discipline of Cybernetics. As such, it draws from various models and concepts within a certain tradition of Cognition [14,131,132]. The authors competently discuss systems interacting with other systems, introducing a Theory of Cybernetic Orders that can be well-aligned with the concept of fractal description in Complex Systems, especially in cognitive ones [133]. In this context, the authors reference the concept of Simplexity, much like Cohen and Stewart presented it as a means to model situations as simply and effectively as possible. To quote Yolles and Fink directly: “Simplex models have a fundamental substructure onto which superstructure is erected. It is the superstructure that is responsible for generating model Complexity and epistemic content” [128]. Moreover, the two authors propose orders of simplex models: “Like squeezing a lemon to get its juice, higher simplex orders conceptually 'compress' complex situations more densely while extracting more meaning from them. This occurs through the use of higher-order conceptual constructs that improve the comprehension, diagnosis, and resolution of stubborn problematic issues”. An example of a simplex variable is *autopoiesis* which provides another way of seeing socio-technical systems [14].
• **Biological Simplexity**: it represents the last recognized nuance of meaning I. In the niche of biological research, this nuance of meaning has found the most suitable conditions for its dissemination, undergoing a true *adaptive radiation* in such context. The key contribution is represented by Alain Berthoz's book “La Simplexité” [56]. Alain Berthoz openly acknowledges his inspiration from the concept proposed by Goldschmidt, but he significantly expands upon it and qualitatively formalizes it into six principles: 1) *The Principle of Refusal*, by which Inhibition, a key trait in living organisms and the human brain, is a significant discovery in evolution. It facilitates competition, decision-making, adaptability, and stability. Various brain centers, such as the cerebellum, basal ganglia, and prefrontal cortex, employ inhibition for coordinating actions, predicting outcomes, and decision-making [134]. This enables humans to distance themselves from reality, modify their perspectives, and exercise executive functions to suppress primitive cognitive strategies and reflexes. Thinking, creating, and acting all involve inhibition and disinhibition, fostering critical examination, akin to the concept of "bracketing" in phenomenology by Edmund Husserl [135]; 2) *The Principle of Specialization and Selection*, which is exemplified by the sensory cues used by different animal species. Each species focuses on cues vital for its survival, such as the tick relying on the smell of butyric acid and heat to find hosts [85]. This principle applies broadly to decision-making, involving the selection of relevant information for action, aligning with the idea of parsimony seen in various domains [55,136]. This selection is not limited to stimulus-response processes but involves adopting a perspective where the brain acts as a comparator and emulator. Attentional mechanisms play a role in this filtering, and modularity within the brain, dedicated to various processes, is crucial. Humans, uniquely, have the capacity to transcend their *Umwelt* to some extent, allowing them to create alternative perspectives; 3) *The Principle of Probabilistic Anticipation*, such principle involves anticipation based on memory, combining prospective and retrospective elements to contextualize the present within a changing universe. This process allows for comparing sensory data with past actions and predicting future consequences [137]. The thalamus, which processes sensory information, demonstrates this dual control. Anticipation based on memory necessitates probabilistic functioning in the face of uncertainty, as seen in fields like Robotics using the Kalman filter. Researchers often apply Bayesian inference, derived from Thomas Bayes theorem, to model human processes. Bayes theorem involves assessing the probability of hypotheses being correct based on available information, past memories, and future predictions. Simplexity suggests that complex problems can be addressed through probability and the emergence of order from disorder. This notion aligns with the idea that enriching experiences with variety is a preference of the brain; 4) *The Detour Principle*, which involves introducing accessory Complexity to simplify complex problems. This concept is akin to when some tourist guide recommends taking a detour to visit a worthwhile site or town [138]. For instance, in Robotics, when controlling a robot that needs to catch objects with unknown dynamic properties in a constantly changing environment, complex nonlinear problems can arise [44]. To address this, the robot uses composite variables, combining elements like position, speed, and acceleration, which appears more complex but actually simplifies the control and prediction of dynamic behavior. This detour approach, seemingly intricate, leads to simpler and more efficient system control, illustrating the essence of Simplexity. Another example is computer simulation for complex systems like the Airbus A380 or robotic surgery, where detours involving advanced technology and algorithms simplify processes and enhance precision. Living organisms also employ detours to solve nonlinear problems, leveraging the nonlinear nature of these detours for effective solutions. While detours are one elegant solution, shortcuts also exist in nature; 5) *The Principle of Cooperation and Redundancy*, which introduces the recovery of potentially lost information. Specialization and selection, as discussed in the second principle, lead to the duplication and generation of significant information but also reduce available solutions. Cooperation is vital in this context, as it allows for multiple values for the same variable to mitigate the risk of error. Cooperation involves various ways to assess critical aspects of the relationship between our bodies and the world. For instance, evaluating your speed requires both specialized sensors and information from other sensors to ensure coherence in the brain's estimations. This goes beyond redundancy, extending to fields like perspective (we acknowledged this phenomenon as “Complixity”, see §5.4). Evolution has equipped us with the ability to view our surroundings either egocentrically (based on our current route) or allocentrically (from a global map-like perspective) [63,139]. These complementary perspectives form a type of Simplexity, enabling us to navigate cities, subways, or forests more efficiently. Such detours are also valuable in simplifying complex situations for CEOs or military strategists. Perspective fundamentally helps in making decisions. Decisions offer an alternative to complex realities, such as a surgeon deciding to operate or not, a judge convicting or acquitting, or a stockholder selling or waiting for the market to recover [65]. These choices depend on context, rules, viewpoints, and previous decisions that serve as frames of reference. Such concepts have been conjured by all the corpus documents regarding architectural and artefacts design [140,141]; 6) *The Principle of Meaning*, by which Simplexity, as discussed for living organisms, imbues meaning into simplification, as simplex solutions are driven by intentions, goals, or functions. The foundation of meaning lies in the action itself, with meaning inherent in life rather than being superimposed upon it. Therefore, the concept of Simplexity encompasses the idea of meaning, and developing a theory of Simplexity involves redefining meaning to include the fundamental role of intended or desired actions. This last principle seems to embrace a kind of finalistic perspective which in potential can be interpreted sensu Weick [142]. According to Berthoz, Simplexity is a theory of meaning as happens for Weick's sensemaking theory, and indeed, this has not escaped the attention of authors who are involved in sensemaking, semiotics, and biosemiotics [57,118]. Berthoz has certainly been the author who has contributed the most to resonate the concept of Simplexity, which has given it the autonomous dignity of a unit of meaning and has most stimulated the imagination of those involved in the cognition of agents, whether biological or socio-technical. He himself has referred to the previous six principles as an invitation to discuss the concept of Simplexity, acknowledging that they may not be definitive or exhaustive. Certainly, they have contributed to adding depth to the nuance of meaning I.

The ultimate outcome of the entire DSR path of knowledge is a set of brand-new constructs, among which stand out the Complexity Simplicities (Simplexity and Complixity), the Content/Context tension, and the Complexity Compression.

## 5 Discussions

The constructs under discussion here are the outcome of the process followed in steps 3.5, 3.6, and partially in 3.7. These artefacts were conceptualized by equally considering contributions from various research strands (§ 4.2) with the multifaceted goal of establishing a common ground where the different facets of Complexity sciences can converge. This, in turn, aims to promote the transferability of concepts from one domain to another (e.g., from a theory of mind to a theory of socio-technical organizations – both considered as entities with agency and cognition), thereby enhancing the depth and generality of reflections within applied Complexity Sciences (e.g., Resilience Engineering).

### 5.1 The Complexity Scientist's point of view

Reality is neither inherently simple nor complex; a car is a relatively simple object from the perspective of Newtonian mechanics, but it becomes less so when viewed through the lens of subatomic mechanics. It is the observer who perceives elements of both Simplicity and Complexity that coexist continually. “Everything said is said by an observer” [14].

On one hand, due to our inherent cognitive limitations, we yearn for Simplicity. On the other hand, we realize its value only when contrasted with Complexity. When we observe reality, we tend to separate a figure from a background, a unit from its environment, content from context, but to quote John Maeda: “What lies in the periphery of simplicity is definitely not peripheral” [143]. If we then delve into this content, its parts become new content residing in a different context. In this manner, reality hierarchically organizes itself into content and context that contain other content, and so on [[144], [145], [146]]. Either Norman, another notorious Design Science authority, argues for the necessity of designing systems that not only confront intrinsic complexity but also render it manageable and comprehensible to users, thereby enhancing user experience and system performance [147].

We tend to think in terms of systems containing other systems, which contain others in a reduction ad absurdum, but defining these systems always involves an observer. Reality is a continuum. However, once these systems are defined, they exhibit a hierarchical structure with superstructures and substructures in a fractal manner [148].

Maurice Yolles and Gerhard Fink, two scholars delved in Cybernetics, distinguished Simplexity in cyber-systems by retrieving the idea of substructure and superstructure. Any model is recursively organized around substructures onto which superstructures are erected [129].

We can observe reality by dismantling layer after layer of this structure, opening one substructure after another, as traditional Cartesian-Newtonian science does – through Reductionism. Alternatively, we can observe the same reality by aggregating portions of context to content, moving upward towards the higher superstructure, in a direction opposite to that taken by Reductionism – as Emergentism does.

At a certain level of this hierarchical description, some simple rules can generate chaos in the higher level, but at the same time, elements of Simplicity also emerge. Even if it were possible to provide a description of a level where only Complexity or only Simplicity exists, at the immediately higher level, both would inevitably emerge.

Let's consider Conway's “Game of Life” [149,150]. It is a simple deterministic system, designed to function through simple rules (no Complexity is present at the level of description coinciding with the source code). At a higher level of observation (i.e., during runtime execution), complex behaviors emerge, exhibiting ordered structures in geometry that display regular patterns despite being dynamically hard to predict, almost *alive*. Everything was already written in the source code. Everything only manifests during execution. In a session of the Game of Life, Simplicity and Complexity coexist.

### 5.2 The tension between content and context

The term “Reductionism” carries the connotation that Complexity at a given level is reduced to simple rules at the lower level. Descending from substructure to substructure, the description of these simple rules becomes increasingly mathematical, as exemplified by Newton's Laws [95,151]. This is a significant advantage when it comes to building economical and efficient predictive models. In the absence of a theory or mathematical law, the process of data acquisition from experimental observation and the use of such data to formulate predictions become crucial, as highlighted, for instance, in the approach used in machine learning [152]. However, the amount of data required for decent predictions in such cases is often huge.

«Information gets drowned in data» [95]. In contrast, if one can have a theoretical mathematical model, only a few pieces of information are sufficient to predict an entire class of phenomena with reasonable accuracy [153]. For instance, given Newton's Laws and specific initial conditions, one can precisely calculate the position of an object in phase space (i.e., position and velocity). Consequently, once the so-called “laws of nature” are established, a coherent explanatory chain traces back from lower levels. In practice, according to this approach, rules and regularities at the n-th level generate behaviors at the (n+1)-th level. With this approach, data can be compressed into theoretical formulations, and these, in turn, can be condensed into meta-theoretical formulations [154]. A few abstract general rules condense data corresponding to many specific cases. The ultimate goal of a reductionist is a comprehensive theory of reality that encompasses all aspects.

Cohen and Stewart do not consider the concept of Holism suitable for indicating the complementary process of examining reality [95]. Such an epistemological position is shared by the entire research team because Holism refers to a system understood as a unit, but this unit is only distinguishable by context. Without a context, there is no unit to observe [15,155] Without a living being, there is no Umwelt nor Ambience [84]. Observing a system without a context is a legacy of Reductionism, e.g., as happens for isolated system in Carnot's machine engineering [156,157]. Cohen and Stewart propose the term “Contextualism”, which integrates the context into the scientist's perspective. In this case as well, a hierarchy of rules, meta-rules, meta-meta-rules (…) manifests, but this time extending outward.

**Fig. 7:**
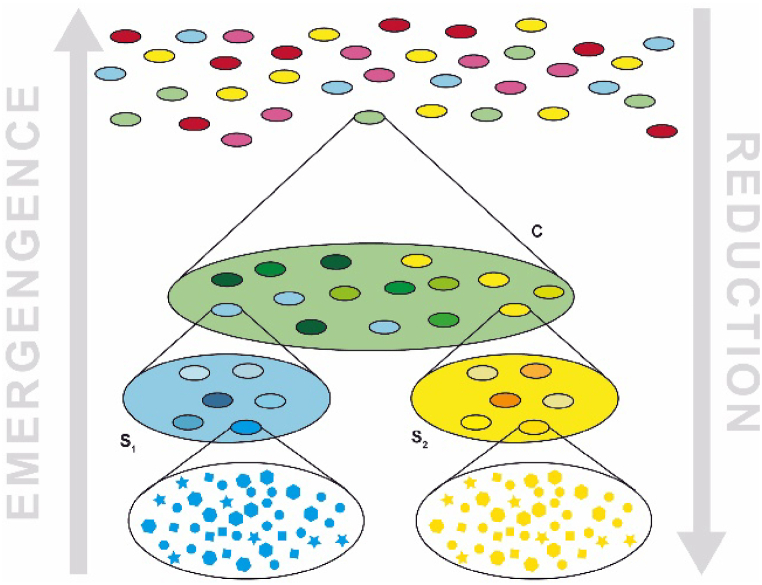
Sketch illustrating a less simplified model of reality in respect to Fig. 1. Any cone is still an aggregation of unities which can be, in turn, aggregated in other unities; the grey arrows are still representing the opposite directions undergone by Reductionism (arrow pointing downward on the right) and Emergentism (arrow pointing upward on the left), but now they are integrated in the same perspective on reality; Following the direction pointed out by Reductionism, Complexity's Simplicities can be discovered dismounting items in simpler pieces, and then studying those pieces' behavior: if they are real simple rules (i.e., patterns, regularities) they will be in force in superstructures as well (e.g., Newton's laws for any simplified bodies); Following the opposite direction different emergent Complexity's Simplicities can be taken into account: whenever a Simplicity emerges shaped by a context defined within the same structural unity we are witnessing a Simplexity (S1 and S2), but when a Simplicity emerges in a novel context which is the envelope resulting from the interaction of different structural unities we are in presence of a Complixity (C). In the latter case, the potential of emergent Simplicities and Complexities is broader. Certainly, this simplified model only scratches the surface of reality, but indeed it successfully grasps the potentiality of combining CAS and/or artefacts, which it a desirable feature for a DSR tool.

If Reductionism is nothing more than the process that interprets the hierarchy of systems in the direction from the current level (n) to the level below (n−1), Emergentism is the process that interprets the world in the direction from the level (n) to the level above (n+1), broadening the context [158], as sketched in Fig. 7. Newton's laws are reductionist; Darwin's theory of evolution is contextualist [95,159]. It explains the Complexity instantiated in different living species not in terms of their genome (which would be a reductionist approach) but in terms of their coevolution over time in a changing environment (the context).

Following this line of thought, the ISO 9241–210:2010 standard emphasizes the critical role of understanding and designing for the relationship between content and context in the creation of user-centered interactive systems [160]. This standard advocates for a thorough grasp of the context — which includes the user, their tasks, and the surrounding environmental conditions — to effectively shape the content. This ensures that the functionalities and information presented are not only relevant but optimally support the user's tasks within their specific operational environment. While reductionism aims to simplify complexity by distilling it into fundamental laws, the design principles advocated by this ISO standard aim to simplify interaction by intelligently adapting the system's content to fit its intended context. This approach, similar to the contextualism suggested by Cohen and Stewart, enhances the user's interaction with the system by aligning it with the dynamism of their real-world environment. Integrating content and context in system design serves to reduce the cognitive load on the user, enhancing both the usability and efficiency of the system, ultimately leading to a more intuitive and productive user experience.

### 5.3 Sub-structure reductionist Simplicities

The simple rules (i.e., the laws of nature) identified by idealizing behaviors in the substructures of the hierarchy of reality (e.g., frictionless models, perfectly Newtonian fluids, homogeneous and isotropic continua, etc.) represent the Simplicity that remains after the neglected Complexity has been accounted for [95].

### 5.4 Super-structure emergent Simplicities (Simplexity and Complixity)

Simplexity is the emergence of simple characteristics at the current level of the hierarchy of reality, as a direct consequence of the high and non-linear interaction of rules, i.e., sub-structural Simplicities. This emergence is independent of the Complexity and fine structure of the underlying level. It occurs when sub-structural rules interact within a limited space of combinations.

When different sub-structural rules – because they belong to entirely different sub-structural units – interact, a different type of Simplicity is observed, which Cohen and Stewart defined as “Complicity”. In an expanded space of possibilities, the contact between previously distinct elements can combine and create new dynamics of interaction. We will clarify the difference between these two types of Simplicity with examples, but for now, it's essential to stress that the second type is generated through a different dynamic and, therefore, deserves to be consolidated into a distinct construct. For this purpose, we have chosen the neologism *Complixity*, signifying both its proximity to Simplexity and its contextual origin, while avoiding potential semantic ambiguities.

The remarkable aspect of reality is not its Complexity. Simplicity can be considered the exceptional fact, thus representing the essence of Emergentism. Not all emergent phenomena are Simplicities, but the emergent phenomena that define self-adaptive complex systems (e.g., living beings, socio-technical systems) are Simplicities.

The Simplicity that emerges at a particular superstructure when the corresponding substructure engages in intra-sub-structural interactions is Simplexity. Berthoz's principle of refusal, similar to Husserlian *epoché*, is an operation for filtering information at the current level, operating on the content regardless of the context [161]. Similarly, the principle of meaning, which condenses a purposive perspective of systems to which agency is attributed, is a principle of Simplexity [162,163]. Regularities (i.e., Simplicities) obtained through the simplified view offered by Reductionism, e.g., Newton's laws of mechanics – applicable to idealized versions of reality where perfectly homogeneous and isotropic spheres exist – work equally well with spherical systems, whether they are atoms or aggregates of atoms. In this case, the fine structure of reality is not necessary to comprehend its general organization [164,165].

When interactions occur between different substructures, nonlinear combinability leads to an explosion of possible emergences. The principle of specialization and selection – the selection of relevant information for action – is simplex as long as the biological species remains within its own Umwelt. However, when a species incorporates elements from a new context, for example, by transcending its Umwelt, it becomes complix [166].

In general, simple behavioral patterns – both Simplexity and Complixity – that we observe are the result of contextual constraints. Regardless of the Complexity of individual units, the context shapes similarly simple behaviors. For example, the presence of a fluid is a contextual regularity to which different animal and plant species can adapt through solutions like wings or fins, conceptually analogous [167]. In unicellular microorganisms, similar metabolic functions are expressed in response to environmental stress factors of varying intensities, regardless the complexity level of community structure [47,168,169]. In higher organisms, cellular respiration requires the combustion of oxygen. Depending on the pressure values at which they operate (i.e., context), some species can advantageously use hemocyanin instead of hemoglobin as a carrier [170]. The emergent function is the same and is not dependent on the internal Complexity of organisms implementing it (e.g., invertebrates vs. vertebrates), but the medium used is different.

In a socio-technical context, an example of similar artefact solutions resulting from a similar context constraint is represented by talking drums and radio. Regardless of the technological content of the artefact that implements it, long-distance articulated communication is based on the frequency modulation of a wave, mechanical in the former case and electromagnetic in the latter. You can change the nature of the medium, but the physics of waves operates in the same way.

### 5.5 Complexity compression and Informative Simplicities

Reinterpreting the model proposed by Yolles and Fink, any substructure is characterized by its own information dispersed into a sea of noise. But higher simplex orders *compress* Complexity and at the same time succeed in extracting more meaning. This can be extremely important for a theory of artefacts: Higher order conceptual constructs improve the comprehension and resolution of substructural Complexity by expanding context (conversely reducing – as they remark by citing Maturana and Varela – *the assumption of all things being equals* [14]. In fact, Simplex and Complixes solutions ascending the hierarchy of the realm discriminate more and more information, while concurrently compress data.

**Fig. 8:**
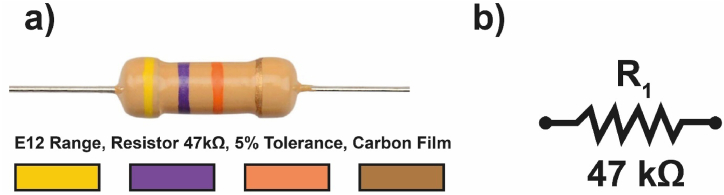
The artefact depicted in panel (a) hides the Complexity concerning many cultural and scientific knowledge, ranging from hundreds of experiments to Maxwell's equations; the color code compresses all such Complexity in an emergent Simplexity, from which further superstructures are erected. It is possible to develop a complex artefact: a symbolic representation (b). The sciences of the artificial continuously leverage similar Complexities by including graph theory and conservation laws in the context.

Indeed, every artefact, whether conceptual or material, compresses Complexity by encapsulating the results of millennia of cultural, technological, and scientific advancement. Consider, for example, what it implies to purchase a 47 kΩ resistor: such a technological object presupposes, even if we can filter it out, the experiences condensed in the so-called Ohm's law, the concept of electrical power, and all the knowledge related to electromagnetism (Fig. 8-a).

Furthermore, on that resistor, we can construct a symbolic object (i.e., a higher-level artefact) like the one in Fig. 8-b, which, due to Complixity, enables efficient and effective methods for circuit design. These designs, in turn, can be compressed into chips, CPUs, and motherboards. When combined with other technologies (expanding the context), they allow us to create increasingly sophisticated, more abstract objects. To manipulate them effectively, one must be able to forget the nature at the original substructural level. A CAD/CAE designer expects to ignore the binary encoding of the underlying electrical voltage value that underlies the entire process. They aim to disregard two centuries of Engineering. The concept of compressing Complexity, as seen in the reinterpreted model by Yolles and Fink, echoes the principles laid out in the ISO 9241-11 standard for usability in visual display terminals (VDTs) [27]. Both frameworks emphasize the importance of managing vast amounts of information through layered, simplified structures. In the case of artefacts, whether conceptual or material, higher-order constructs reduce substructural complexity by encapsulating essential knowledge into manageable forms, much like how glass cockpit systems integrate complex flight data into streamlined displays for pilots. In glass cockpits, information is presented in a way that balances simplicity and complexity, ensuring usability by minimizing cognitive load, allowing users to engage with higher-level abstractions without being overwhelmed by underlying complexities. The symbolic representations and complex artefacts emerging from this compression serve as efficient tools for navigating intricate systems, just as well-designed cockpit interfaces organize and simplify crucial data for pilots.

The significance of robust ergonomic criteria in the inspection of complex systems cannot be overstated, especially as we aim for a balance between technological complexity and operational simplicity. A pertinent study in this domain demonstrated that ergonomic criteria, when carefully applied, not only enhance the detection of usability flaws but also foster a more collaborative and consistent evaluation process among designers. This experimental study, involving thirty-one HSI designers assessing two simplex systems, highlights the criteria's reliability and validity, reinforcing our call for their broader adoption and further refinement in the field of human-system integration [66,171]. Such findings underscore the need for evolving our ergonomic approaches to keep pace with the increasing complexity of socio-technical systems.

Every artefact possesses a potential for applications that depends on the social context. When different socio-technical systems come into contact or when different artefacts do, their non-linear interaction reveals a new potential that cannot be achieved by the separate components. Just as Simplicities, behaviors, and patterns emerge, new potentials also emerge. When Graph Theory, Electrical Engineering, Information Theory, and the HTML protocol combine, the possibilities increase immeasurably. The internet is an example of Complixity. Without the myriad of texts persisting on the web, without neural networks, without vector algebra, the current generative models of natural language (e.g., ChatGPT) wouldn't have the conditions for their existence. The idea of Complexity compression implemented by emergent Simplicities (i.e., Simplexity and Complixity) can integrate both the Berthozian *perçaction* and the simultaneous execution of Simplicity in action and Complexity in thought [65,118]. Similarly, the activity of constructing meaning through combinatorial rules from elementary structural items in potentially inexhaustible articulations, a result of Simplexity and Complixity, encompasses data deconvolution [172], cognitive explanations of language [173,174], organizational systems [142], and entire social organization [39,175].

The conceptual artefacts here proposed can indeed enable new interpretations and potential further developments of existing artefacts, whether idiographic or nomothetic. For example, the FRAM is known as a methodological tool for mapping portions of socio-technical systems [176,177]. It is notoriously difficult to read in the case of maps that are more than just trivial. In the article “Unveil key functions in socio-technical systems: mapping FRAM into a multilayer network”, the authors complement the method with techniques to compress Complexity without information loss (sensu Colville) and propose a framework for theorizing alternative FRAM instantiations to identify resilient mitigation actions in the socio-technical system under study [178]. This activity becomes much clearer when expressed in terms of Complixity: the FRAM analyst generates these new instantiations by expanding the context each time.

In another context, the authors have proposed a formal framework (i.e., WAx) for mapping knowledge transfers between different varieties of work in cyber-socio-technical systems [179]. The fractal nature of WAx and the socio-technical agents included in it can be easily interpretable in terms of Simplexity and Complixity. For this purpose, the authors, when asked about it, found Yolles and Fink's fractal framework of simplex structures and substructures particularly convenient [130]. Apart from this initial outline, the idea has not been further developed but appears to be particularly promising.

The relationship between Complixity and Anticipation – one of the four pillar abilities proposed by Hollnagel [26] – might be particularly significant to Resilience Engineers. In fact, Anticipation can be explained as a Complix effect resulting from the comparison between fictious Responding activity and actual lessons learnt, i.e., hypothetical contexts projected into the realm of potential and the observed contexts in the historical record (i.e., Learned). It is only by comparing what one is experiencing with situations already experienced and situations that could be experienced – thus comparing potential contexts – that Anticipation can be operationalized. The clarity of such idea represents – per se – a novel outcome in Resilience Engineering.

Finally, the need to identify formulations capable of adequately accounting for complex systems (not only CAS) is also felt in the field of Physics. For example, a very recent article published in the Proceedings of the National Academy of Sciences expresses the same need [86]. Although it is an independent work conducted with substantially different methods and different purposes, it presents surprising similarities in its conclusions. This only serves to strengthen and substantiate both research efforts, thus providing initial evidence of the validity of the proposed constructs.

## 6 Conclusions

The present research has successfully demonstrated the applicability of the latest DSR methods to the construction of conceptual artefacts, managing to integrate in a structured manner methodologies – such as Grounded Theory – coming from an apparently distant tradition from Engineering. The nomothetic definitional constructs (*N Def*) of Simplexity, Complixity, and their implications in terms of Complexity compression show the potential to further advance Complexity applied disciplines such as Resilience Engineering.

We do not yet know what possibilities at the super-structure level may arise, but without clarification of the dynamics of the different Simplicities operating in complex self-adaptive systems, it will not be possible to progress further in the study of anthills, nor in the study of organizational phenomena. Neuroscience scholars as much as designers, cognition theorists as much as resilience engineers are equally in need of these artefacts to foster an emergent Complixity.

By following step by step the specific guideline suggested by Akoka, we succeeded in our initial intent. An extensive documentary analysis was used as a database. It was our only source of information, with the exception of the research group's expertise, which is the major limitation of this work. It must be marked again that, when examined within the framework of various cultural contexts, the constructs of Complexity and its Simplicities can be significantly influenced by cultural, linguistic and societal factors. Within specific societal settings, individuals may readily embrace the idea of “Simplexity” without explicitly categorizing it as such. In our literature review, we explicitly referenced the term “Simplexity”, therefore, potentially excluding articles that discuss the concept of Simplexity without using the term itself. This limitation, however, presents an opportunity for further studies in the field. Furthermore, the results were validated limited to the research group. The dynamics of Anticipation, a pillar ability for resilient systems, can be fruitfully reinterpreted under the lens of Simplicities. Existing artefacts, both concrete and intangible, such as resistors and WAx (or FRAM), can be reimagined leveraging both Simplexity and Complixity in their processes. The final effectiveness of the conceptual artefacts defined in this research deserves more investigation. This is a task we leave to subsequent works. Only the acceptance by the academic community and the successful application in designing further artefacts will reveal their ultimate value. As authors, we shall resolve the pursue of Akoka's G3 guideline by continuing the present research matching the path of knowledge we just undertook.

The exploration of Simplexity and Complixity within complex systems opens a captivating gateway to understanding the intricate dynamics of our world. This research not only illuminates the paradoxical nature of Simplicity coexisting with Complexity, but also underscores the fundamental role of these principles in diverse fields, from technical systems to social sciences. As we continue to dissect the subtleties of Complexity, new paradigms will emerge, reshaping our approach to complex systems. This journey promises to empower us with insights that transcend disciplinary boundaries. Only fostering a deeper comprehension of the interconnected world in which we live, we can offer innovative solutions to the challenges we face.

## CRediT authorship contribution statement

**Andrea Falegnami:** Writing – review & editing, Writing – original draft, Visualization, Validation, Supervision, Software, Resources, Project administration, Methodology, Investigation, Funding acquisition, Formal analysis, Data curation, Conceptualization. **Andrea Tomassi:** Writing – review & editing, Writing – original draft, Visualization, Validation, Supervision, Software, Resources, Project administration, Methodology, Investigation, Funding acquisition, Formal analysis, Data curation, Conceptualization. **Chiara Gunella:** Writing – review & editing, Resources, Investigation. **Stefano Amalfitano:** Writing – review & editing, Resources, Investigation. **Giuseppe Corbelli:** Writing – review & editing, Resources, Investigation, Formal analysis. **Karolina Armonaite:** Resources, Investigation. **Claudio Fornaro:** Writing – review & editing, Resources, Investigation. **Luigi Giorgi:** Writing – review & editing, Writing – original draft, Resources, Investigation. **Alessandro Pollini:** Investigation, Conceptualization. **Alessandro Caforio:** Writing – review & editing, Supervision, Methodology, Investigation, Formal analysis, Conceptualization. **Elpidio Romano:** Writing – review & editing, Validation, Supervision.

## Ethics and consent to participate

Human participants in this study are co-authors of this paper and provided their consent to participate by contributing to the manuscript**.**

## Availability of data and materials

Data included in article/supplementary material is referenced in the article. The datasets generated and/or analyzed during the current study are available from the corresponding author on reasonable request.

## Declaration of competing interest

The authors declare that they have no known competing financial interests or personal relationships that could have appeared to influence the work reported in this paper.
