# Supplementary material for: Defining conceptual artefacts to manage and design simplicities in complex adaptive systems
Source: Heliyon. 2024 Dec 9;10(24):e41033. doi: 10.1016/j.heliyon.2024.e41033 (PMC11696777; doi:10.1016/j.heliyon.2024.e41033)
Supplement: Multimedia component 3 [file mmc3.docx]

**Identification of studies via other methods**

**Identification of studies via databases and registers**

Records identified from:

Websites (n = 0)

Organisations (n = )

Citation searching (n = 5)

etc.

Records removed *before screening*:

Duplicate records removed (n = 3)

Records marked as ineligible by automation tools (n = 0)

Records removed for other reasons (n = 0 )

Records identified from*:

Databases (n = 89 )

Registers (n = 0 )

**Identification**

Records screened

(n = 86)

Records excluded**

(n = 0)

Reports not retrieved

(n = 0)

Reports sought for retrieval

(n = 5)

Reports sought for retrieval

(n = 86)

Reports not retrieved

(n = )

**Screening**

Reports assessed for eligibility

(n = 5)

Reports excluded:

Reason (n = 0)

Reports assessed for eligibility

(n = 86)

Reports excluded:

Reason (n = 0)

Studies included in review

(n =86 )

Reports of included studies

(n = 5)

**Included**

*Consider, if feasible to do so, reporting the number of records identified from each database or register searched (rather than the total number across all databases/registers).

**If automation tools were used, indicate how many records were excluded by a human and how many were excluded by automation tools.

*From:*  Page MJ, McKenzie JE, Bossuyt PM, Boutron I, Hoffmann TC, Mulrow CD, et al. The PRISMA 2020 statement: an updated guideline for reporting systematic reviews. BMJ 2021;372:n71. doi: 10.1136/bmj.n71.
